# PP04 Extrapolating Survival Curves In Oncology: Advancing Economic Modeling To Meet Next-Generation Health Technology Assessment Demands

**DOI:** 10.1017/S0266462325102249

**Published:** 2025-12-29

**Authors:** Stéfani Borges, Mateus Cerqueira, Gustavo Frio, Bruna Santos, Ivan Zimmermann

## Abstract

**Introduction:**

Oncology economic modeling often struggles with estimating clinical benefits like overall survival (OS) and progression-free survival (PFS), especially with short-duration trials involving extrapolating survival curves. This study analyzed methods used by the Brazilian National Committee for Health Technology Incorporation (CONITEC) in health technology assessment (HTA) to adapt economic models to evolving healthcare needs.

**Methods:**

A descriptive analysis was conducted on the economic evaluations from CONITEC’s recommendation reports on oncology drugs published between January 2019 and December 2023. Three independent reviewers extracted data on outcomes, survival curve distributions, validation methods, and time horizons. The study focused on identifying patterns in modeling practices and explored whether these methods integrated with the expanding scope of HTA, including the use of non-traditional data sources and adaptive techniques.

**Results:**

Thirty CONITEC reports were analyzed, evaluating 32 oncology treatments across 45 indications. The most common economic models were partitioned survival analysis (PartSA) (58%) and Markov models (33%). The Weibull distribution was the most frequent in OS extrapolations (50% for PartSA; 84% for Markov). PFS extrapolations showed a preference for exponential distribution in PartSA (35%) and Weibull in Markov models (54%). Methods for model validation were reported in 69 percent of Markov models and 100 percent of PartSA models. Extrapolation horizons ranged from 10 years (75% of models) to 30 years (31%).

**Conclusions:**

The analysis highlighted the reliance on traditional models, such as PartSA and Markov, for modeling long-term cancer progression. However, these models may not fully capture treatment complexity. Emerging methods and the integration of diverse data sources, such as real-world data, are vital for improving HTA relevance and addressing the evolving demands of healthcare systems and next-generation evidence.

